# Differentiation of pulmonary sclerosing pneumocytoma from solid malignant pulmonary nodules by radiomic analysis on multiphasic CT

**DOI:** 10.1002/acm2.13154

**Published:** 2020-12-28

**Authors:** Xiao‐Qiong Ni, Hong‐kun Yin, Guo‐hua Fan, Dai Shi, Liang Xu, Dan Jin

**Affiliations:** ^1^ The Second Affiliated Hospital of Soochow University Suzhou China; ^2^ Beijing Infervision Technology Co.,Ltd Beijing China

**Keywords:** CT, PSP, radiomic analysis, SMPN

## Abstract

**Purpose:**

To investigate the diagnostic value and feasibility of radiomics‐based texture analysis in differentiating pulmonary sclerosing pneumocytoma (PSP) from solid malignant pulmonary nodules (SMPN) on single‐ and three‐phase computed tomography (CT) images.

**Materials and Methods:**

A total of 25 PSP patients and 35 SMPN patients with pathologically confirmed results were retrospectively included in this study. For each patient, the tumor regions were manually labeled in images acquired at the noncontrast phase (NCP), arterial phase (AP), and venous phase (VP). The least absolute shrinkage and selection operator (LASSO) method was used to select the most useful predictive features extracted from the CT images. The predictive models that discriminate PSP from SMPN based on single‐phase CT images (NCP, AP, and VP) or three‐phase CT images (Combined model) were developed and validated through fivefold cross‐validation using a logistic regression classifier. Model performance was evaluated using receiver operating characteristic (ROC) analysis. The predictive performance was also compared between the Combined model and human readers.

**Results:**

Four, five, and five features were selected from NCP, AP, and VP CT images for the development of radiomic models, respectively. The NCP, AP, and VP models exhibited areas under the curve (AUCs) of 0.748 (95% confidence interval [CI], 0.620–0.852), 0.749 (95% CI, 0.620–0.852), and 0.790 (95% CI, 0.665–0.884) in the validation dataset, respectively. The Combined model based on three‐phase CT images outperformed the NCP, AP, and VP models (all *p* < 0.05), yielding an AUC of 0.882 (95% CI, 0.773–0.951) in the validation dataset. The Combined model displayed noninferior performance compared to two senior radiologists; however, it outperformed two junior radiologists (*p* = 0.004 and 0.001, respectively).

**Conclusion:**

The Combined model based on radiomic features extracted from three‐phase CT images achieved radiologist‐level performance and could be used as promising noninvasive tool to differentiate PSP from SMPN.

## 1 Introduction

Pulmonary sclerosing pneumocytoma (PSP) is a rare benign tumor originating from undifferentiated respiratory epithelium.^1^, ^2^ As the basis for diagnosis of PSP, imaging physicians currently consider the morphological characteristics of an oval‐shaped, well‐defined, smooth boundary and the tail sign as distinguishing hallmarks^3^, ^4^; however, these characteristics are restricted by subjectivity and unsatisfactory reproducibility. Moreover, it is challenging to distinguish PSP from solid malignant pulmonary nodules (SMPN) when these nodules fail to exhibit malignant computed tomography (CT) signs such as spiculation, pleural indentation, and lobulation. Thus, using only on visual characteristics may easily lead to misdiagnosis and thus cause the most effective treatment period to be missed.

Radiomic analysis is an emerging technique that can noninvasively reflect tumor heterogeneity by extracting high throughput of quantitative features on images, and it has shown a strong application value in differentiation, efficacy evaluation, and prognosis judgment in oncology, especially in distinguishing primary lung cancer from inflammatory nodules. However, most conventional studies of radiomic analysis only focus on noncontrast CT images.^5^, ^6^, ^7^ To the best of our knowledge, there are few studies concerning the use of radiomic analysis to differentiate PSP from SMPN, especially by adding contrast‐enhanced imaging. The purpose of this study was to investigate the diagnostic value and feasibility of radiomics‐based texture analysis in differentiating PSP from SMPN without malignant CT signs on single‐ and three‐phase CT images.

## 2 Materials and Methods

### 2.1 Patients enrollment

This retrospective study was approved by our ethics committee board. Data for patients who underwent chest contrast‐enhanced CT between January 2010 and March 2020 were initially retrieved. All patients underwent biopsy or surgery, and the tumor type was pathologically confirmed. The inclusion criteria were as follows: (1) all patients had undergone preoperative CT scans with noncontrast phase (NCP), arterial phase (AP), and venous phase (VP) within 2 weeks before surgery; (2) isolated solid pulmonary nodules larger than 1 cm; (3) nodules without cavitation and satellite lesions; and (4) no performance of radiotherapy or chemotherapy. The exclusion criteria were as follows: (1) solid pulmonary malignant tumors with obvious malignant CT signs such as lobulated shape, spicules of margin, or pleural indentation and (2) pure ground‐class nodules and mixed ground‐glass nodules. Ultimately, 25 PSP patients and 35 SMPN patients (including lung adenocarcinoma, lung squamous carcinoma, small cell lung cancer, or lung metastases) were enrolled for further analysis.

### 2.2 CT image acquisition

Contrast‐enhanced chest CT examinations were performed using a GE Discovery CT750 HD CT scanner (GE Healthcare, Princeton, NJ, USA). The CT scanning parameters were as follows: 120‐kV tube voltage, 360‐mA tube current, 0.6‐sec tube rotation time, 512 × 512 matrix, SFOV large body, and 5‐mm section thickness. All CT images were reconstructed using a 0.625‐mm slice thickness.

For the contrast‐enhanced CT scan, patients were injected with 1.5 mL of iodine (300 mg I/mL) by a pump injector at a rate of 3 mL/s into the antecubital vein. Images of arterial and venous phases were obtained at a postinjection delay of 5.7 sec and 30 sec after initiation of contrast material injection, respectively.

### 2.3 Image analysis

All CT images were manually labeled by a radiologist with more than 10 years of experience. The pixel‐wise tumor regions were segmented on the maximal slice of CT images using ITK‐SNAP version 3.8.0 (http://www.itksnap.org). Contouring was carefully drawn within the borders of the tumors while avoiding covering the adjacent bronchi and vessels. The segmentation results were reviewed and modified by another senior radiologist with more than 20 years of experience. Both radiologists were blinded to pathologic results.

### 2.4 Radiomic feature extraction and selection

Feature extraction and selection was performed using the InferScholar platform version 3.3 (InferVision, Beijing, China). The PyRadiomics package (version 2.1.2, https://github.com/Radiomics/pyradiomics) was used to automatically extract radiomic features from the tumor regions of CT images. A total of 991 nonzero features were extracted, including first‐order intensity statistics features (n = 234) and texture features (Gray Level Dependence Matrix [n = 182], Gray Level Co‐occurrence Matrix [GLCM, n = 273], Gray Level Size Zone Matrix [GLSZM, n = 94], and Gray Level Run Length Matrix [GLRLM, n = 208]).

To avoid overfitting and reduce model complexity, dimension reduction of the features was conducted using the two‐sample t test and the Least Absolute Shrinkage and Selection Operator (LASSO) approach. The differential features between PSP and SMPN groups in the training dataset were firstly selected, and then the most valuable radiomic features (those most closely associated with the discrimination between PSP and SMPN) were chosen for further analysis.

### 2.5 Development of radiomic models

We developed three single‐phase based predictive models using radiomic features extracted from the CT images of the noncontrast phase (NCP model), arterial phase (AP model), and venous phase (VP model), respectively. A Combined model incorporating radiomic features of the three phases was also constructed. A logistic regression (LR) classifier was used to discriminate PSP from SMPN patients. The PSP and SMPN groups were defined as positive and negative in the classification process, respectively. LR was a statistical modeling technique where the probability of a category was related to a set of explanatory variables.^8^ The logistic model was defined by the following equations:

(1) $$z=a_{0}+\sum_{i=1}^{n}a_{i}x_{i}$$

(2) $$P\left(z\right)=\frac{e^{Z}}{1+e^{Z}}$$

where Z was a measure of the contribution of the explanatory variables *x_i_* (*i* = 1, …, n), *a_i_* represented the regression coefficients obtained by maximum likelihood in conjunction with their standard errors △*a_i_*, and *P*(*z*) was the categorical response of the variables. The models were trained using the scikit‐learn toolkit, and the parameters were as follows: c = 1, penalty = ‘l2’, tol = 0.0001, solver = libninear; other parameters were set by default. To better train our models and build them more robustly based on a limited sample size, the fivefold cross‐validation method was applied.

The development and validation of all models were performed using InferScholar platform version 3.3 (InferVision, Beijing, China).

### 2.6 Assessment of radiologists

Preoperative CT images from noncontrast and contrast CT scan were retrospectively reviewed by four radiologists (two senior radiologists with more than 20 years of experience each, and twos junior radiologists with 4 and 5 years of experience in thoracic imaging), then made a judgment between PSP and SMPN. The judgment criterion was according to the diagnostic experience that mainly included size, shape, internal density, strengthening mode, and tumor periphery. Radiologists were unaware of the patients' clinical information and pathologic results.

### 2.7 Statistical analysis

The receiver operating characteristic curve was used to evaluate the capacity of the predictive models for the discrimination of PSP from SMPN tumors in the training and validation datasets, with respect to sensitivity, specificity, and the area under curve (AUC). Sensitivity was defined as TP / (TP + FN), specificity was defined as TN / (FP + TN), where TP, FP, FN, and TN refer to true positive, false positive, false negative, and true negative, respectively.

The Mann–Whitney U test was used for evaluation of the differences in continuous variables with a distribution across categories. The association between categorical variables in different groups was accessed using the chi‐squared test. Delong’s test was applied for comparing differences between two or more AUCs of different models. All tests were two sided, and a *P* value < 0.05 was considered statistically significant. All analyses were performed using Prism 5 for Windows (Version 5.01) and MedCalc (Version 18.11.3).

## 3 Results

### 3.1 Study design

From January 2010 to March 2020, a total of 60 patients (23men, 37 women)were retrospectively enrolled in this study according to inclusion and exclusion criteria. The age for the 25 patients with PSP was 52.0 ± 11.42 years (range, 24–68 years) and 62.7 ± 11.17 years (range, 17–77 years) (*P* < 0.05) for the 35 patients with SMPN. The NCP, AP, VP, and Combined models using different phase of CT images were conducted using the fivefold cross‐validation method.

### 3.2 Feature selection

**Fig. 1:**
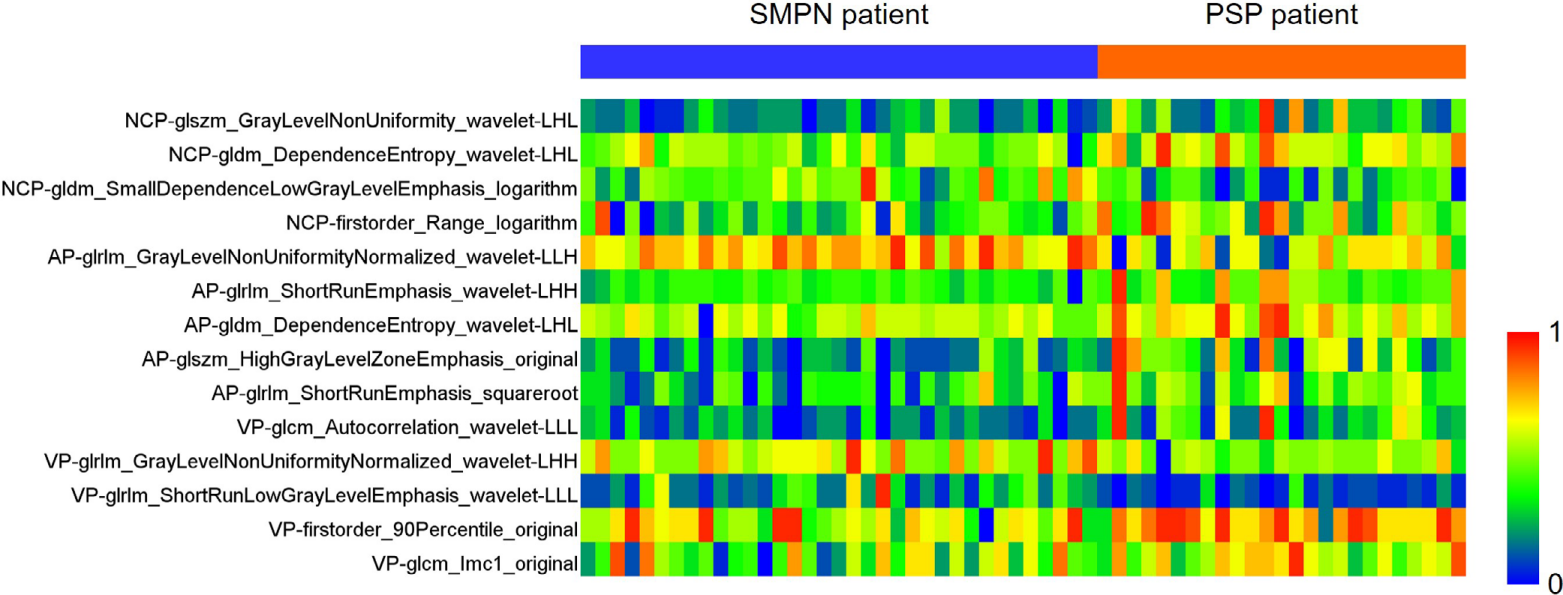
Heatmap of selected radiomic features from noncontrast phase, arterial phase, and venous phase images. Each row represents a radiomic feature, and each column corresponds to one patient (separately grouped for PSP and SMPN patients).

A two‐step method was applied for feature selection. There were 353, 398, and 379 features obtained from the NCP, AP, and VP of CT images after feature selection with a two‐sample t test, respectively. These key features were further selected using LASSO regression. Finally, four, five, and five features selected from the NCP, AP, and VP of CT images were used for the development of radiomic models, respectively. The feature heatmap was plotted according to the normalized radiomic feature values (Figure 1).

### 3.3 Development and validation of the radiomic models

**Fig. 2:**
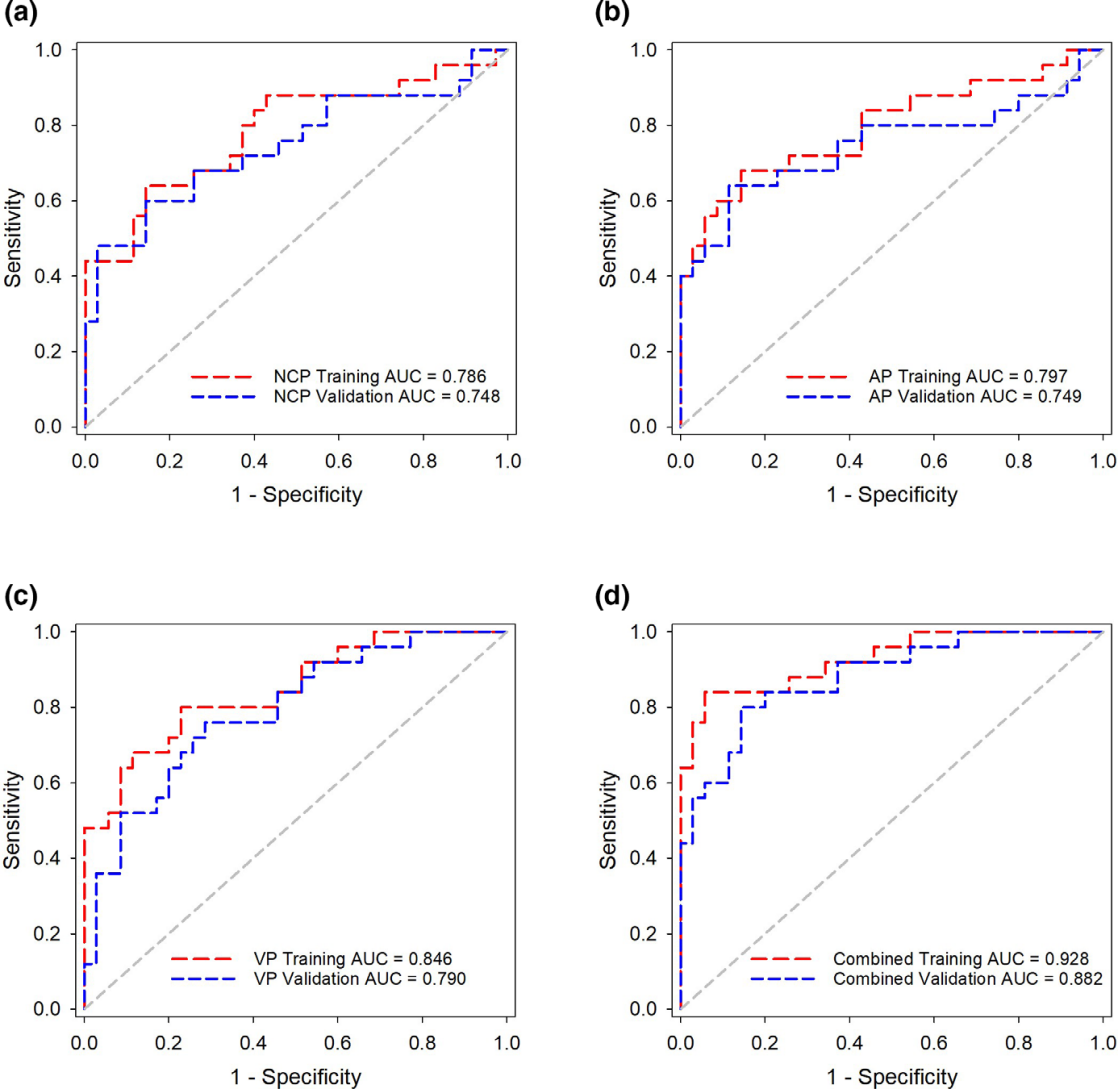
ROC curves of the (a) NCP model, (b) AP model, (c) VP model, and (d) Combined model in the training and validation datasets, respectively.

The diagnostic performance of the radiomic models was evaluated using the receiver operating curve (ROC) analysis in the validation dataset. As shown in Figure 2 (Figure 2), the AUCs of NCP, AP, VP, and Combined models were 0.786 (95% CI, 0.661–0.882), 0.797 (95% CI, 0.673–0.890), 0.846 (95% CI, 0.729–0.926), and 0.928 (95% CI, 0.831–0.979) in the training dataset, respectively. The NCP, AP, VP, and Combined models exhibited AUCs of 0.748 (95% CI, 0.620–0.852), 0.749 (95% CI, 0.620–0.852), 0.790 (95% CI, 0.665–0.884), and 0.882 (95% CI, 0.773–0.951) in the validation dataset, respectively.

**Fig. 3:**
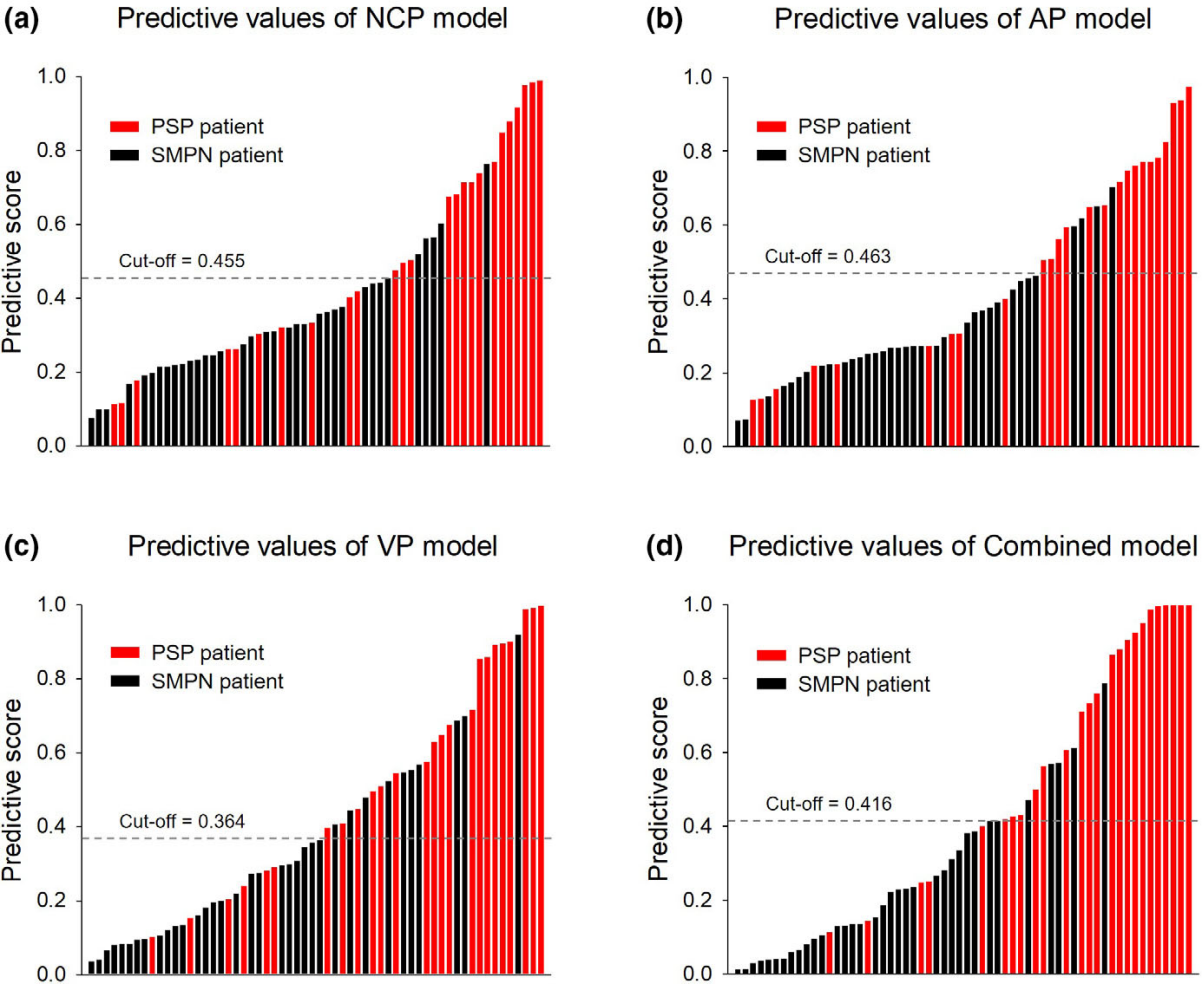
The diagnostic score of the NCP, AP, VP, and Combined models for each patient in the validation set. Red bars represent the scores for PSP patients, while black bars represent the scores for SMPN patients.

The Combined model showed better performance than the other single‐phase models (Delong’s test, *P* = 0.022 vs the NCP model, *P* = 0.039 vs the AP model, *P* = and 0.038 vs the VP model). The accuracy, sensitivity, specificity, and AUCs of these predictive models are summarized in Table 1. The predictive scores of the NCP, AP, VP, and Combined models for each patient in the validation dataset are shown in Figure 3 (Figure 3).

**Table 1:** Diagnostic performance of the predictive models in the validation dataset.

Models	Cut‐off	Sensitivity (95% CI)	Specificity (95% CI)	AUC (95% CI)
NCP model	0.455	60.0% (38.7%–78.9%)	85.7% (69.7%–95.2%)	0.748 (0.620–0.852)
AP model	0.463	64.0% (42.5%–82.0%)	88.6% (59.9%–89.6%)	0.749 (0.620–0.852)
VP model	0.364	76.0% (54.9%–90.6%)	71.4% (53.7%–85.4%)	0.790 (0.665–0.884)
Combined model	0.416	80.0% (59.3%–93.2%)	85.7% (69.7%–95.2%)	0.882 (0.773–0.951)

Abbreviations: AUC, area under the receiver‐operating characteristic curve; NCP, noncontrast phase; AP, arterial phase; VP, venous phase.

### 3.4 Comparison of diagnostic efficiency between the Combined model and radiologists

**Fig. 4:**
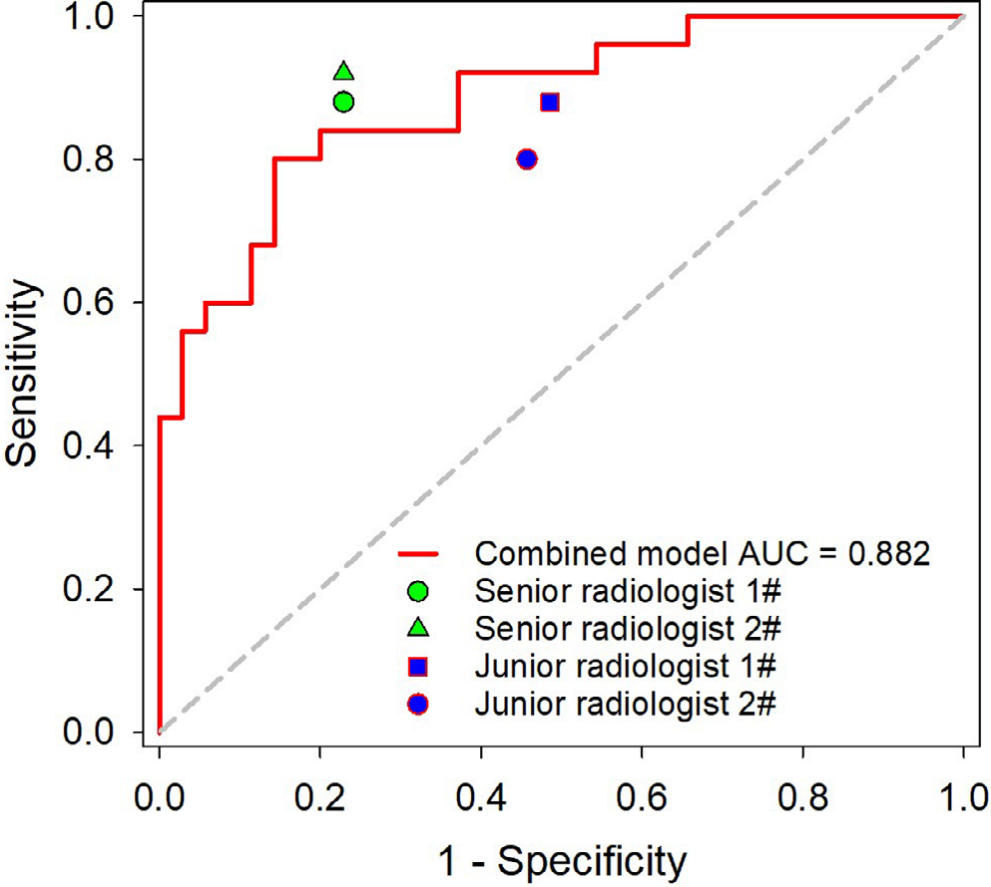
Comparison of the Combined model and radiologists in differentiating PSP from SMPN.

ROC analysis results suggested that the Combined model was noninferior to radiologists in differentiating PSP from SMPN. Delong’s test indicated that the Combined model achieved similar diagnosis capability to the two senior radiologists (*P* = 0.275 and 0.521, respectively), and the model significantly outperformed the two junior radiologists (*P* = 0.004 and 0.001, respectively). The detailed performance of the Combined model and radiologists are summarized in Table 2; the ROC analysis is shown in Figure 4.

**Table 2:** Diagnostic performance comparison of the Combined model and radiologists.

Models	Sensitivity	Specificity	AUC (95% CI)	*P* value (vs the Combined model)
Combined model	80.0% (20/25)	85.7% (30/35)	0.882 (0.773–0.951)	N/A
Senior radiologist 1	88.0% (22/25)	77.1% (27/35)	0.826 (0.706–0.911)	0.275
Senior radiologist 2	92.0% (23/25)	77.1% (27/35)	0.846 (0.729–0.926)	0.521
Junior radiologist 1	88.0% (22/25)	51.4% (18/35)	0.697 (0.565–0.809)	0.004*
Junior radiologist 2	80.0% (20/25)	54.3% (19/35)	0.671 (0.538–0.787)	0.001*

Abbreviations: AUC, area under the receiver‐operating characteristic curve.

* *P* < 0.05.

## 4 Discussion

PSP is a subtype of adenoma and is derived from a dual population of surface cells resembling type II pneumocytes and round cells. Histologically, the tumor is solid, papillary, sclerotic, or hemorrhagic.^9^, ^10^ Most studies have shown that benign tumors usually exhibit a progressive and homogeneous enhancement pattern.^2^, ^11^ Notwithstanding, Cheung et al. reported that the inhomogeneous enhancement pattern was dominant because of differences in histopathological predominant types.^12^ In addition, Chung et al. showed that the morphologic features or enhancement patterns in CT images could not help distinguish between lung cancer and PSP.^13^ In addition, PSP also has potentially malignant potential, such as lymph node metastasis, slow‐growing multiple nodules, and pleural dissemination, which has been frequently reported despite its rarity.^14^, ^15^, ^16^ Therefore, it is difficult to distinguish PSP from pulmonary malignancies using only conventional imaging features, especially malignant nodules or masses without malignant signs.

Radiomics‐based CT texture analysis is a technique that can carry out mathematical analysis and operation on the pixels, voxel gray levels, and spectral characteristics in the images, then quantify the heterogeneity of tumor tissue structure through specific texture parameters.^17^, ^18^, ^19^ CT texture analysis can provide an objective assessment of lesion and organ spatial heterogeneity such as cellular density, angiogenesis, and necrosis; this analysis can provide information beyond that of conventional subjective image assessment.^20^ In addition, in some aspects, the information gained is also beyond that of random sampling biopsy, as biopsy analysis only evaluates a small part of the tumor, while texture analysis reflects the tumor as a whole.^21^

In present study, we found that the VP model showed higher sensitivity and discrimination capability than the other two single‐phase models, and the discrimination capability was further improved in the Combined model by incorporating features from three‐phase CT images than the NCP, AP, and VP models (all *P* < 0.05), yielding an AUC of 0.882. The NCP model showed inferior discrimination capability compared to that of the other single‐phase models. The results from our study differ from previously published texture analysis studies. For instance, Dennie et al. reported that noncontrast‐enhanced CT exhibited a higher specificity (88%) and AUC (0.9) than that of contrast‐enhanced CT (38% and 0.6, respectively) in the differentiation of primary lung cancer and granulomatous nodules.^22^ The reasons for the inconsistency of results are not yet clear. However, some studies manifested that using IV contrast medium may obscure textural information and influence the results, because contrast‐related factors such as speed of infusion, amount of contrast agent, image‐related factors (scan and delay time), and patient‐related factors (cardiac output, anatomical differences) are not standardized and contribute to inconsistent results.^23^ Nonetheless, our results are in accord with other texture analysis studies. For example, Son et al. reported that iodine‐enhanced imaging could improve efficacy of diagnosing invasive adenocarcinoma from AIS or MIA compared to diagnosing using nonenhanced imaging from 0.888 to 0.959, respectively (*P* = 0.029).^24^ Based on previous reports,^25^, ^26^ we speculate that the reason may be the difference in intratumoral microvessel density between benign and malignant tumors, namely, the difference of capillary perfusion and permeability could be more prominent after contrast administration.

The present results also showed that the Combined model achieved noninferior performance to that of two senior radiologists, without an obvious statistical difference, but that the model outperformed two junior radiologists. Thus, application of the radiomic model may be conducive for junior radiologists to use in discriminating PSP and SMPN. Tumor peripheral characteristics such as the vascular bordering sign or vascular correction sign, which relate to discriminating PSP and SMPN, were not extracted into the radiomic model; this may be why the Combined model provided no significant advantage over senior radiologists.

Some limitations of this study should be noted. First, this was a retrospective study from a single center and with a small sample size. We will conduct prospective multicenter studies with a larger sample size to avoid bias in modeling and results and increase its repeatability. Second, radiomic features were only extracted inside the tumor in our study, while some radiomic features at the tumor periphery related to discriminating PSP and SMPN were not extracted; further studies should focus on integrating the radiomic features inside and outside the tumor to improve the diagnosis abilities. Third, clinical data were not analyzed in combination with radiomic features, and these should be included into further study for nomogram analysis.

## 5 Conclusion

In conclusion, we established a radiomic model based on multiphasic CT in differentiating PSP from SMPN on single‐ and three‐phase CT images, and the results showed that models based on three‐phase CT images achieve better performance than those using single‐phase CT images. The results manifested that radiomics‐based texture analysis could serve as a promising non‐invasive tool for radiologists to differentiate PSP and SMPN.

## AUTHORS’ CONTRIBUTIONS

Xiao‐Qiong Ni was involved in drafting the work, acquisition, and analysis and interpretation data for the work. Hong‐kun Yin was involved in analysis and interpretation of data for the work. Guo‐hua Fan was involved in revising it critically for important intellectual content. Dai Shi was involved in acquisition and analysis data for the work. Liang Xu* was involved in conception or design of the work, revising it critically for important intellectual content. Dan Jin* was involved in conception or design of the work, acquisition data for the work and final approval of the version to be published.

## Conflict of Interest

No authors have any conflict of interest to disclose.
